# Clinical Causes of Inflammation in Peritoneal Dialysis Patients

**DOI:** 10.1155/2014/909373

**Published:** 2014-05-06

**Authors:** Yeoungjee Cho, Carmel M. Hawley, David W. Johnson

**Affiliations:** ^1^Department of Renal Medicine, University of Queensland at Princess Alexandra Hospital, Ipswich Road, Woolloongabba, Brisbane, QLD 4102, Australia; ^2^Translational Research Institute, Woolloongabba, Brisbane, QLD 4102, Australia; ^3^Department of Nephrology, Princess Alexandra Hospital, Level 2, ARTS Building, Ipswich Road, Woolloongabba, Brisbane, QLD 4102, Australia

## Abstract

Inflammation at both systemic and local intraperitoneal levels commonly affects peritoneal dialysis (PD) patients. Interest in inflammatory markers as targets of therapeutic intervention has been considerable as they are recognised as predictors of poor clinical outcomes. However, prior to embarking on strategies to reduce inflammatory burden, it is of paramount importance to define the underlying processes that drive the chronic active inflammatory status. The present review aims to comprehensively describe clinical causes of inflammation in PD patients to which potential future strategies may be targeted.

## 1. Introduction

Peritoneal dialysis (PD) is a form of home-based renal replacement therapy for patients with end-stage kidney disease (ESKD) that uses a patient's peritoneum as a dialysis membrane across which water and solutes (e.g., electrolytes and glucose) are exchanged between dialysis fluid and blood. PD has several advantages including greater ease of technique to master, greater preservation of residual renal function (RRF), early survival advantage, and superior cost effectiveness compared to haemodialysis [1–3]. Greater preservation of RRF is significant as it leads to a better technique survival by enhancing PD adequacy and ultrafiltration capacity [4].

Despite these benefits, the outcome of PD patients remains poor and cardiovascular events (CVE) continue to be the leading cause of death in PD patients [5]. Higher CVE burden in chronic kidney disease (CKD) patients compared to those without CKD is astounding (proportion of patients without CVE 38.7% versus 61.7%) [6]. Moreover, the relative risk of death is paradoxically higher in CKD patients identified as the “lower” risk group (i.e., younger patients or those with a lower prevalence of CVE [7]), supported by data from the United States Renal Data System [8]. In contrast to the general population, advances in medical therapy for patients with CVE (e.g., aspirin, lipid-lowering agents) have not decreased the CVE-related burden in patients with ESKD. An increase in the delivery of dialysis dose has not translated into a mortality benefit in PD patients [9]. Additional risks have been attributed to the presence of nontraditional risk factors, such as inflammation, which have been shown to promote proliferation and infiltration of inflammatory cells into the tunica intima of small arteries, leading to the development of atherosclerosis and stenosis [10]. An association between a decline in RRF in patients with CKD and progressively increased level of systemic inflammatory burden which is most marked in those receiving renal replacement therapy, such as haemodialysis, has been well established [11, 12]. At present, there is no clear evidence to suggest any significant difference in the systemic inflammatory burden based on the type of dialysis modality received (i.e., haemodialysis versus peritoneal dialysis) [13].

## 2. Defining Inflammation and Its Relevance

**Figure 1:**
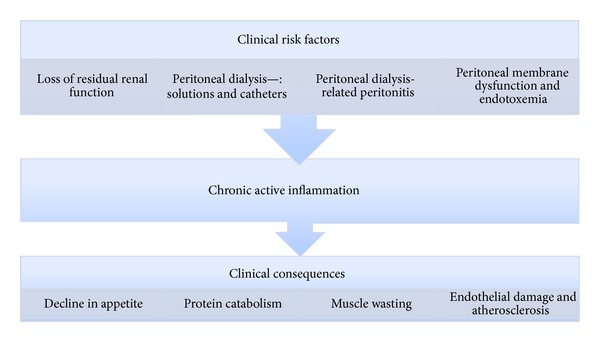
Flow diagram demonstrating clinical risk factors of inflammation in peritoneal dialysis patients leading to chronic active inflammation and clinical consequences.

Inflammation can be defined as a localised protective response elicited by injury or destruction of tissues that serves to destroy, dilute, or sequester both the injurious agent and injured tissue. Hence, it is a physiological response and in the form of an acute response to infections, trauma, or toxic injury, it helps the body to defend against pathophysiological insults. However, if inflammation becomes prolonged and persistent in the form of the so called chronic acute-phase reaction, it may lead to adverse consequences, such as decline in appetite, increased rate of protein depletion in skeletal muscle, hypercatabolism, endothelial damage, and atherosclerosis [14–19] (Figure 1).

There are several markers that can be measured to gauge the level of inflammatory burden, such as C-reactive protein (CRP). CRP levels can rise rapidly and markedly in response to acute inflammatory stimulus from increased synthesis by hepatocytes to contribute to host defense and innate immune response [20]. Its induction in hepatocytes in turn is regulated by cytokines such as interleukin-6 (IL-6), which is a pleiotropic immunomodulatory cytokine that plays a critical role in many innate and acquired inflammatory processes [21]. Dysregulation of IL-6 signalling has been implicated in a variety of chronic disease pathologies and in immune and inflammatory diseases [21]. However, the activities of these proinflammatory cytokines depend on the involved cell types and its microenvironment. For example, after an “acute” injury, tumor necrosis factor-like weak inducer of apoptosis (TWEAK) promotes tissue regeneration by stimulating progenitor cells but in “chronic” diseases where TWEAK is persistently activated it alters tissue repair by inhibiting differentiation of the same progenitor cells [22, 23]. The inflammatory pathways are clearly complex and dependent on many conditions (e.g., acute versus chronic, microenvironment) and therefore are often difficult to clearly characterise.

## 3. Prevalence of Inflammation in PD Patients

In PD patients, inflammation can be broadly compartmentalised into two types, systemic and local intraperitoneal inflammation. As recently reported by the GLOBAL fluid study, these two represent distinct underlying processes that likely require different preventative or therapeutic approaches [24]. The reported prevalence of systemic inflammation measured using CRP ranges between 12% and 65% in PD patients, depending on the cut-off value used to define the level of inflammation [25, 26]. A number of longitudinal studies have also been reported increasing burden of inflammation measured using interleukin-6 (IL-6) with longer time on PD at both systemic and intraperitoneal levels [27–29].

Interest in inflammatory markers as targets of therapeutic intervention has been considerable as they are recognised as predictors of poor patient outcomes (e.g., mortality). However, prior to embarking on strategies to reduce inflammatory burden, it would be of paramount importance to define the underlying causes that drive the chronically inflamed state. The present review aims to comprehensively describe clinical causes of inflammation in PD patients at which potential future therapeutic targets may be aimed.

## 4. Clinical Causes of Inflammation in Peritoneal Dialysis Patients

### 4.1. Uraemia versus Residual Renal Function Loss

A number of studies have reported an association between lowered RRF and higher systemic inflammatory burden in predialysis [11] and dialysis patients [30, 31]. Furthermore, the level of inflammatory cytokines progressively increases with worsening renal function [32]. It remains uncertain as to whether these associations are primarily a result of an impaired renal clearance of inflammatory cytokines, direct stimulation of cytokine generation by uraemic milieu, or simply a consequence of adverse effect of inflammation on RRF.

The importance of renal elimination of proinflammatory cytokines was described using animal models where the half-lives of injected interleukin-1 (IL-1) [33] and tumour necrosis factor (TNF) [34] were increased after nephrectomy. In addition, preclinical studies have demonstrated pathogenic mechanisms of uraemic toxins on inducing proinflammatory cytokine production and renal tubular cell injury via nuclear factor-kappa*β* (NF-*κβ*) [35] and oxidative stress pathways [36], respectively. The direct stimulation of systemic inflammatory burden by uraemic toxins was further supported by the findings from a recent cross-sectional observational study of 149 chronic kidney disease (CKD) patients (mean eGFR 40 ± 9 mL/min/1.73 m^2^; range 25–59 mL/min/1.73 m^2^) which showed that serum uraemic toxin levels (i.e., indoxyl sulphate) were significantly and independently associated with serum IL-6, TNF-*α*, and interferon-*γ* (IFN-*γ*) concentrations (unpublished) [37].

Nonetheless, the relationship between RRF and inflammation becomes less clear once patients commence dialysis due to the presence of dialysis-specific factors (e.g., peritonitis) that can stimulate systemic inflammatory cytokine production independent of the background RRF decline. In fact, the GLOBAL fluid observational study involving 959 PD patients from 10 centres in Korea, Canada, and the United Kingdom did not observe any significant association between patients' residual urine volume and systemic IL-6 concentrations in their prevalent (*P* = 0.7) or incident cohorts (*P* = 0.3) [24]. Similarly, a biomarker substudy of the balANZ trial was not able to demonstrate the presence of any statistically significant association between the loss of RRF and serum IL-6 concentrations over the 24 months of follow-up period in the 175 incident PD patients (*P* = 0.27) [29]. In contrast to these reports, Chung and colleagues described an association between a greater loss in RRF and higher serum CRP concentrations (≥10 mg/L) after 12 months of PD in incident patients (*P* < 0.05) [15]. Some of the differences in observed outcomes could have resulted from dissimilar statistical analysis techniques (e.g., continuous versus categorical data analyses) and the inflammatory marker measured (IL-6 versus CRP).

Similarly, the impact of RRF on intraperitoneal inflammation remains unclear due to conflicting reports from published literature. A previous peritoneal biopsy study has observed significantly worse peritoneal membrane injury in patients with uraemia (predialysis) compared to those with normal renal function (*P* = 0.01) [38]. Therefore, it is plausible that the uraemic milieu itself may promote the extent of peritoneal injury and better preserved RRF may lower the intraperitoneal inflammatory burden associated with peritoneal injury. The GLOBAL fluid study reported significantly lower levels of dialysate IL-6 with a higher urine volume in their prevalent cohort (coefficient −0.1 per litre, *P* = 0.01) but not in incident cohort (coefficient 0.03 per litre, *P* = 0.2) [24], whereas the balANZ trial observed no significant association between rate of RRF decline with dialysate IL-6 concentrations (*n* = 88, *P* = 0.67) [28]. Conclusions that can be drawn from these studies were however limited by the absence of longitudinal data [24] and relatively small sample size [28, 29] which could have lowered the statistical power to detect differences in outcome. Therefore, at present, it remains uncertain as to what the true implication of RRF loss is, for systemic and local inflammatory burdens in PD patients. It is likely that RRF has some role in influencing these levels, but its impact may be overshadowed by the presence of other competing factors, such as infections or repeated exposures to PD solutions. Perhaps some of these questions can be better answered through future studies evaluating the relationship between presence of uraemic toxin levels and inflammatory markers in PD patients.

### 4.2. Peritoneal Dialysis

The cumulative and progressive nature of peritoneal membrane injury with longer PD duration has been well documented [38]. Conventional PD solutions are characterised by their acidic pH (5.0–5.8), high lactate concentrations (75.5–214 mmol/L), high osmolality (320–520 mOsm/kg), and contamination by glucose degradation products (GDP) and have been shown to contribute to adverse outcomes demonstrated in preclinical studies [39–41]. Repeated exposures to conventional PD solutions [38] and peritonitis episodes [42] contribute to peritoneal injury, which in turn is an important cause of local inflammation with resultant adverse functional outcomes, such as higher peritoneal solute transport rate (PSTR) [43–45]. Indeed, dialysate IL-6 concentration has been identified as the most reliable predictor of PSTR by a number of single centre studies and has now been substantiated by the large multicentre GLOBAL fluid study [24, 27, 46]. IL-6 is secreted in large quantities by peritoneal mesothelial cells in response to inflammatory stimuli and is modulated by exposure to PD solutions [47]. An increase in intraperitoneal IL-6 concentrations with longer PD duration (i.e., at 24 months) was consistently demonstrated by extension studies of the Balnet trial (biocompatible 57.6 ± 54.5 pg/mL versus 143 ± 69.6 pg/mL, *P* < 0.001; standard 47 ± 31.2 pg/mL versus 121 ± 69 pg/mL, *P* < 0.001) [48] and the balANZ trial (median 7.22 pg/mL versus 31.35 pg/mL, *P* < 0.001) [28]. Similar results were yielded in the peritonitis-free cohort of the balANZ trial (*n* = 56, *P* < 0.001) [28].

In contrast to these consistent results pertaining to the relationship between PD duration and intraperitoneal inflammation, there are contradicting reports about the impact of PD duration on systemic IL-6 concentrations. In a single-centre, retrospective observational study of incident PD patients (*n* = 31) receiving treatment using conventional PD solutions, Pecoits-Filho and colleagues described a significant increase in plasma IL-6 concentrations from baseline to one year (median 3.7 pg/mL versus 6.5 pg/mL, *P* < 0.05) [27]. Similar results were observed from a substudy of the balANZ trial (*n* = 175) at 24 months (*P* = 0.006) [29]. The GLOBAL fluid study however described a longer PD duration as a significant predictor of a random plasma IL-6 level in prevalent (coefficient 0.02 per year; *P* = 0.04) but not in incident PD patients (coefficient −0.2 per year; *P* = 0.4) [24]. Furthermore, a prospective observational study (*n* = 109) reported a lack of significant change in serum IL-6 concentrations over twelve months [46]. Although the reasons for such discrepant findings are unclear, some of the differences may stem from variations in the study design, differences in assay techniques and samples (serum versus plasma) used to measure IL-6 levels, and the duration over which these changes were measured. Furthermore, whereas intraperitoneal inflammation is mainly driven by PD-related factors, such as repeated exposures to PD solution or peritonitis, systemic inflammation can be additionally influenced by many “PD-independent” factors such as systemic infection that could have affected the observed outcomes.

### 4.3. Potential Role of “Biocompatible” Peritoneal Dialysis Solutions

A recent report by Ayuzawa and colleagues [49] suggests that some of peritoneal membrane injury from PD can be minimised by using PD solutions that are more “biocompatible”. Consequently, over the past two decades, the PD solutions that are more “biocompatible” have been manufactured. Minimisation of GDP formation has been achieved through development of the multicompartment bag system, which allows for heat sterilisation and storage to occur at a lower pH [50]. Moreover, a bicarbonate-buffer system has been used to lower exposure to lactate. Several preclinical studies have demonstrated that use of these solutions has resulted in improved cytokine profiles and cellular function, including the host immune system [51–56]. Therefore, the use of these “biocompatible” PD solutions may lead to changes in the intraperitoneal environment with the potential benefits of decreasing the level of intraperitoneal inflammatory burden and improving peritoneal membrane function (i.e., PSTR).

Indeed, Cho and colleagues in their prospective observational study involving 187 incident PD patients described an increase in PSTR in patients receiving standard solutions over 12 months unlike those treated using biocompatible solutions who maintained a stable PSTR [46]. However, this study suffered from a relatively high proportion of patient drop-outs (41.1%) and the choice of therapy (biocompatible versus standard) was at the discretion of each patient's treating physician, thereby introducing a risk of selection bias. More importantly, the study did not report whether there were any differences in the dialysate IL-6 concentrations between patients who received standard versus biocompatible PD solutions.

Over the past few years, several RCTs conducted to examine differences in clinical outcomes from the use of biocompatible PD solutions have not been able to demonstrate a reduction in dialysate IL-6 levels with its use [57–59]. To date, only one study conducted by the bicarbonate/lactate study group reported a significant decrease in dialysate levels of IL-6 in patients who received biocompatible PD solutions (*n* = 61) compared to conventional PD solutions (*n* = 31) over 6 months (*P* = 0.01) [60]. However, the strength of conclusions that can be drawn from these studies was restricted by large drop-out rates (>20%) [57], risk of carry-over effects due to cross-over design [58], and a lack of accounting for the confounding effect of peritonitis [57–60].

More recently, the GLOBAL fluid study and a substudy of the balANZ trial explored the impact of biocompatible PD solutions use on dialysate IL-6 concentrations and found no significant difference based on the type of PD solutions received [24, 28]. Comparable results were yielded when analyses were repeated in the peritonitis-free cohort (*n* = 56) [28]. The results from these studies were also however challenged by several limitations including lack of detailed examination of the history of biocompatible PD solutions exposure in the study participants (i.e., patients indicated as using biocompatible PD solutions could have been treated with conventional PD solutions prior to study entry) [24], analysing data in a cross-sectional manner [24], the risk of selection bias, and a small sample size (*n* = 88) [28]. Therefore, at present, based on a generally suboptimal level of evidence, there is no convincing effect of biocompatible PD solutions use on decreasing the level of dialysate IL-6.

The use of biocompatible PD solutions may theoretically decrease the inflammatory burden at a systemic level by lowering the extent of peritoneal injury and GDP-mediated nephrotoxicity leading to residual renal function decline [61]. Szeto and colleagues (*n* = 50) were the first to present the data demonstrating an improvement in systemic inflammation levels, as evidenced by lower serum CRP measurements, in patients using biocompatible PD solutions at 12 months (1.77 ± 0.42 mg/L versus 7.73 ± 2.42 mg/L, *P* = 0.03) [62]. However, several RCTs comparing the effect of biocompatible PD solutions to standard PD solutions on systemic IL-6 concentrations have not been able to demonstrate any differences between patients receiving biocompatible or standard solutions [29, 48, 58, 59, 63]. Although the lack of difference observed between the two groups could have resulted from relatively short follow-up (i.e., <12 months) [58], cross-over study design [58], inclusion of biocompatible PD solutions with higher GDP content [59, 63], small sample size, or a large drop-out rate [29], it could be a real phenomenon. Therefore at present, based on the best available evidence, in spite of a demonstrated beneficial effect on maintaining stability of PSTR, the use of biocompatible PD solutions does not appear to lower the burden of inflammation at both systemic and intraperitoneal levels.

### 4.4. Peritoneal Dialysis Catheters

Whilst the majority of the literature has attributed morphologic and functional changes of the peritoneal membrane to PD solutions and peritonitis, the PD catheter itself can also induce peritoneal inflammation independently with associated disruption of peritoneal membrane integrity [64, 65]. Certainly, the development of biofilm bacterial growth in PD catheters due to skin bacteria [66] and PD peritonitis episodes [67] is well acknowledged and can lead to dissemination of bacteria into the PD fluid with resultant peritonitis [68]. However, there are reports of proinflammatory responses associated with the use of PD catheters independent of bacteria-related biofilm. For instance, Flessner and colleagues described amplification in the peritoneal inflammatory response and peritoneal membrane injury in rodent models when they administered low-GDP bicarbonate-buffered solution via catheters compared to needle-injection over a 20-week study period [69]. They also observed formation of a sterile inflammatory cell layer (i.e., biofilm) within the catheter lumen, which they proposed as a source of proinflammatory cascade. Although the applicability of their findings to humans remains questionable, these results raise questions about the role of PD catheters in promoting inflammation in PD patients.

### 4.5. Peritoneal Dialysis-Related Peritonitis

PD-related peritonitis is an important source of inflammation at both intraperitoneal [70, 71] and systemic levels [72, 73] and contributes to approximately 20% of PD technique failures [74] and 2–6% of deaths [75, 76]. The reported peritonitis rates range between 0.06 and 1.66 episodes per patient-year [77]. PD peritonitis can lead to excessive peritoneal inflammatory responses leading to mesothelial cell injury and thickening of the submesothelium compartment, resulting in peritoneal fibrosis and sclerosis [78]. The severity and extent of peritoneal membrane damage correlate with the number and severity of peritonitis episodes [78]. An elevation in proinflammatory cytokines from PD dialysate samples (e.g., IL-1 and IL-6) is evident from the time of clinical presentation with acute peritonitis and their levels remain significantly elevated for at least 6 weeks after the initial presentation (compared to control patients, *P* < 0.001) [71]. Furthermore, lack of a decrease in dialysate IL-6 concentrations with treatment of acute peritonitis has been shown to predict relapsing peritonitis [70]. Similarly, the onset of peritonitis is associated with an increase in serum CRP levels [72, 73] and higher CRP levels have been associated with worse short-term outcomes (e.g., transfer to haemodialysis) and long-term patient outcomes (e.g., subsequent peritonitis event, all-cause mortality) [72]. Although the adoption of several preventative strategies, such as the use of disconnect (twin-bag and Y-set) systems [79, 80] and preoperative administration of intravenous antibiotics prior to PD catheter insertions [81, 82], has decreased overall peritonitis rates, there remains significant room for further improvement.

### 4.6. Peritoneal Membrane Dysfunction and Endotoxemia

Peritoneal membrane dysfunction can be clinically manifested as inadequate small solute clearance and ultrafiltration failure. Loss of ultrafiltration can in turn lead to the development of volume overloaded state, including the risk of bowel oedema, which can precipitate endotoxemia by promoting translocation of macromolecules from the gut [83]. Other factors that are thought to promote endotoxin translocation in CKD patients include uraemia [84, 85], malnutrition leading to atrophy of intestinal mucosa [86], and constipation through bacterial overgrowth. Bacterial endotoxin is a lipopolysaccharide which makes up the majority of the outer membrane of gram-negative bacteria found in the gut. In CKD patients, significantly higher endotoxin levels were observed amongst patients classified as fluid-overloaded (defined by inferior vena cava diameter adjusted for body surface area >11.5 mm/m^2^) when compared with patients with normal fluid status (0.85 ± 0.11 ng/L versus 0.61 ± 0.05 ng/L, *P* < 0.05) [87]. More importantly, endotoxin is a strong proinflammatory stimulus and endotoxemia has been consistently associated with an increase in the level of systemic inflammation in CKD [88], HD [89], and PD patients [90]. At present, it remains uncertain whether interventions, such as improvement in fluid status or the level of uraemia, can result in a decrease in endotoxemia and systemic inflammation in humans and should be studied in future.

## 5. Other Treatment Options to Reduce the Inflammatory Burden

Beyond the aforementioned possible interventions for reducing inflammation in PD patients (Table 1), there have only been a limited number of studies on treating the chronic inflammatory state in patients receiving PD. These include the use of agents known to possess anti-inflammatory (e.g., statins) [91] or antioxidant properties (e.g., N-acetylcysteine) [92] that resulted in a decreased level of systemic inflammation burden. Others have proceeded with targeted treatment in those diagnosed with clinical significant periodontitis with similar results [93]. Although these outcomes are encouraging, they need to be interpreted with caution as they were relatively small sized studies (largest study *n* = 76) from single-centres and their results have not been validated by others.

## 6. Summary and Future Directions

Inflammation is a common complication of PD patients at both systemic and local (i.e., intraperitoneal) levels. Chronic inflammatory status is associated with a number of clinically significant adverse patient outcomes, including malnutrition, peritoneal membrane dysfunction, and cardiovascular events. Although there are a number of potentially modifiable clinical causes of inflammation, a limited number of intervention studies to date have not been able to successfully identify effective strategies to lower inflammatory burden in this patient group. Future studies should focus on better defining of the pathogenic mechanisms underlying peritoneal and systemic inflammatory cascade in PD patients and evaluating the efficacy of interventions targeting these identified factors.

## Figures and Tables

**Table 1:** Summary of proposed mechanisms underlying clinical causes of inflammation in peritoneal dialysis patients and possible treatment options.

Clinical causes of inflammation	Proposed mechanism	Possible treatment options
Loss of residual renal function	(i) Impaired renal clearance of inflammatory cytokines (ii) Direct stimulation of cytokine generation by uraemic milieu (iii) Adverse effect of inflammation on residual renal function	Strategies to improve residual renal function, such as (i) avoidance of nephrotoxic agents or acute kidney injuries (ii) use of biocompatible PD solutions

Peritoneal dialysis: use of conventional peritoneal dialysis solutions	(i) Cumulative peritoneal membrane injury (ii) Glucose degradation product mediated nephrotoxicity	Use of biocompatible solutions characterised by neutral pH, low glucose degradation product content

Peritoneal dialysis catheters	Biofilm formation within the catheter lumen	Use of catheters resistant to biofilm formation

Peritoneal dialysis-related peritonitis	Induces acute inflammatory response	Peritonitis prevention strategies: (i) disconnect systems (twin-bag and Y-set) (ii) preoperative administration of intravenous antibiotics prior to PD catheter insertion

Peritoneal membrane dysfunction and endotoxemia	Bowel oedema from volume overload precipitating endotoxemia by translocation of macromolecules from gut	Improvement in fluid status
